# Ptychographic X-ray speckle tracking^1^

**DOI:** 10.1107/S1600576720005567

**Published:** 2020-05-29

**Authors:** Andrew J. Morgan, Harry M. Quiney, Saša Bajt, Henry N. Chapman

**Affiliations:** a ARC Centre of Excellence in Advanced Molecular Imaging, School of Physics, University of Melbourne, Parkville, Victoria 3010, Australia; b CFEL, Deutsches Elektronen-Synchrotron DESY, Notkestrasse 85, 22607 Hamburg, Germany; c DESY, Notkestrasse 85, 22607 Hamburg, Germany; d The Hamburg Centre for Ultrafast Imaging, Luruper Chaussee 149, 22761 Hamburg, Germany; e Department of Physics, University of Hamburg, Luruper Chaussee 149, 22761 Hamburg, Germany

**Keywords:** X-ray speckle tracking, ptychography, phase retrieval, wavefront metrology, in-line projection holography

## Abstract

A method is presented for the measurement of the phase gradient of a wavefront by tracking the relative motion of speckles in projection holograms as a sample is scanned across the wavefront. By removing the need to obtain an undistorted reference image of the sample, this method is suitable for the metrology of highly divergent wavefields. Such wavefields allow for large magnification factors that, according to current imaging capabilities, will allow for nanoradian angular sensitivity and nanoscale sample projection imaging. Both the reconstruction algorithm and the imaging geometry are nearly identical to that of ptychography, except that the sample is placed downstream of the beam focus and that no coherent propagation is explicitly accounted for. Like other X-ray speckle tracking methods, it is robust to low-coherence X-ray sources, making it suitable for laboratory-based X-ray sources. Likewise, it is robust to errors in the registered sample positions, making it suitable for X-ray free-electron laser facilities, where beam-pointing fluctuations can be problematic for wavefront metrology. A modified form of the speckle tracking approximation is also presented, based on a second-order local expansion of the Fresnel integral. This result extends the validity of the speckle tracking approximation and may be useful for similar approaches in the field.

## Abstract 2

A method for the simultaneous measurement of a wavefront’s phase and the projection hologram of an unknown sample is presented. This method relies on an updated form of the speckle tracking approximation, which is based on a second-order expansion of the Fresnsel integral.

## 1. Introduction   

New facilities are providing ever more brilliant X-ray sources. To access the full potential of these sources we need X-ray optics that are capable of focusing light to meet the requirements of various imaging modalities. Thus there is an increasing need for at-wavelength and *in situ* wavefront metrology techniques that are capable of measuring the performance of these optics to the level of their desired performance. This is a challenging task, as current X-ray optics technologies are attaining focal spot sizes below 10 nm (Mimura *et al.*, 2010 ▸; Huang *et al.*, 2013 ▸; Morgan *et al.*, 2015 ▸; Bajt *et al.*, 2018 ▸; Murray *et al.*, 2019 ▸). Furthermore, adaptive optics are being employed to correct for wavefront aberrations by altering the physical state of a lens system in response to real-time measurements of wavefront errors (Mercère *et al.*, 2006 ▸; Zhou *et al.*, 2019 ▸). Such systems therefore benefit from fast and accurate wavefront metrology for rapid feedback.

Wavefront metrology techniques generally fall into one of three categories (Wilkins *et al.*, 2014 ▸; Wang *et al.*, 2015 ▸): (i) direct phase measurements, such as interferometry using crystals (Bonse & Hart, 1965 ▸); (ii) phase gradient measurements, such as Hartmann sensors (Lane & Tallon, 1992 ▸), coded aperture methods (Olivo & Speller, 2007 ▸) and grating-based interferometry (David *et al.*, 2002 ▸); and (iii) propagation-based methods sensitive to the second derivative of the wavefront’s phase (Wilkins *et al.*, 1996 ▸; Wang *et al.*, 2015 ▸; Bérujon *et al.*, 2014 ▸).

One such method, falling into the second category above, was introduced by Bérujon, Ziegler *et al.* (2012 ▸) and Morgan *et al.* (2012 ▸) (no relation to the current author). This method is a wavefront metrology technique based on near-field speckle-based imaging, which was termed the ‘X-ray speckle tracking’ (XST) technique. In XST, the 2D phase gradient of a wavefield can be recovered by tracking the displacement of localized ‘speckles’ between an image and a reference image produced in the projection hologram of an object with a random phase/absorption profile. Additionally, XST can be employed to measure the phase profile of an object’s transmission function. Thanks to the simple experimental setup, high angular sensitivity and compatibility with low-coherence sources, this method has since been actively developed for use in synchrotron and laboratory light sources [see Zdora (2018 ▸) for a recent review].

In ptychography, a sample is scanned across the beam wavefront (typically at or near the focal plane of a lens) while diffraction data are collected in the far field of the sample. An iterative algorithm is usually employed to update initial estimates for the complex wavefront of the illumination and the sample transmission functions. If illuminated regions of the sample overlap sufficiently, then it is possible for a unique solution for both of these functions to be obtained (Hüe *et al.*, 2010 ▸). Thus, ptychography is an imaging modality that performs both aberration-free sample imaging and wavefront metrology simultaneously. This is in contrast to XST where these two imaging modalities correspond to separate imaging geometries.

Ptychography can also be performed in the near-field diffraction regime, as reported for example by Nugent *et al.* (1996 ▸). Stockmar *et al.* (2013 ▸) found that the illumination must be sufficiently inhomogeneous to allow for a successful reconstruction, and those authors suggested the use of an additional diffuser as a means of achieving this. Consequently, this near-field ptychographic imaging approach closely resembles that of many XST-based approaches in its experimental configuration. The key distinction here is that, in ptychography, a fully coherent wave model is employed. This can lead to non-unique solutions in situations where an XST-based approach would yield a well defined solution, although typically this solution will contain less information at lower resolution than a successful ptychographic reconstruction.

We propose a combined approach, which we term ptychographic X-ray speckle tracking (PXST). In this approach, near-field in-line holograms are recorded as an unknown sample is scanned across an unknown wavefield. Estimates for the undistorted sample projection image and the wavefield are then updated on the basis of the observed speckle displacements. There is no reference image and no additional speckle-producing object is required. This imaging geometry allows for XST to be used for highly divergent X-ray beams, thus expanding the applicability of this simple and robust method to include next-generation high-numerical-aperture X-ray lenses.

Bérujon *et al.* (2014 ▸) have proposed a similar method, also based on XST and compatible with highly divergent beams. In their approach, the second derivative of the wavefront phase is measured. Additionally, nanoradian angular sensitivity can be achieved with relatively small step sizes in the scan of the sample on a piezo-driven stage (discussed further in the next section). In contrast, PXST more closely aligns with current XST-based techniques, such as the ‘unified modulated pattern analysis’ method of Zdora *et al.* (2017 ▸, 2018 ▸), that do not rely on small sample translations.

In Section 2, we briefly review the XST method and its extension to PXST. In Section 3 we present the governing equation, which is based on a second-order expansion of the Fresnel diffraction integral (presented in Appendix *A* [App appa]). The region of validity for the speckle tracking approximation determines the applicable imaging geometries, which are presented in Section 4 and Appendix *B* [App appb]. We present the iterative reconstruction algorithm and the target function, which is to be minimized by the algorithm, in Section 5. Conditions for the uniqueness of the solution are discussed in Appendix *C* [App appc]. Finally, the theoretically achievable angular sensitivity of the wavefront reconstruction and the imaging resolution of the sample projection image are presented in Appendix *D* [App appd]. For reference, we define the principal mathematical symbols used throughout the paper in Table 1 ▸. In Table 2 ▸, we summarize the main results of this article and refer the reader to the relevant sections.

## 2. Background   

The problem with wavefront metrology is that it is much more difficult to measure a wavefront’s phase than its intensity; the intensity can be measured directly by placing a photon-counting device at any point in the wavefront’s path, whereas the phase information is indirectly encoded in the wavefront’s intensity profile as it propagates through space. For plane-wave illumination, no measurement of the wavefront’s intensity alone will reveal its direction of propagation. One solution to this problem is to place an absorbing object at a known point in the path of the light, from which the direction of propagation can then be inferred from the relative displacement between the centre of the object and the shadow cast on a screen some distance away, just as the angle of the sun can be estimated by following the line from a shadow to its object.

**Figure 1:**
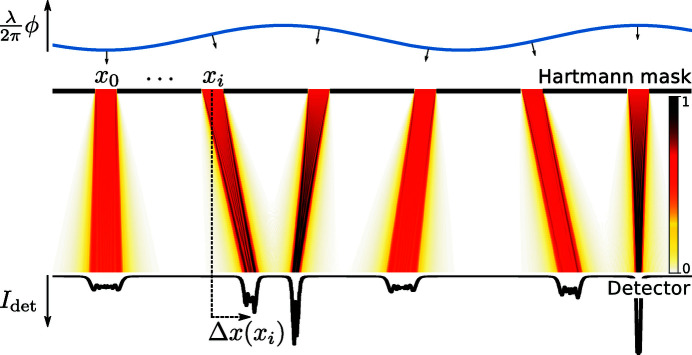
Illustration of the Hartmann sensing principle. Top: phase of the wavefront incident on the entrance surface of the mask. The phase has been scaled by λ/2π so that the normal to the tangent is parallel to the local direction of propagation of the wavefront. The arrows indicate the direction of propagation at the centre of each mask hole. Middle: intensity of the wavefront as it propagates from the mask (top) to the detector (bottom). The colour scale is shown on the right. Bottom: the one-dimensional intensity profile of the wavefront as measured by the detector.

This simple idea forms the basis of the Hartmann sensor (Daniel & Ghozeil, 1992 ▸), shown schematically in Fig. 1 ▸. Originally designed to measure aberrations in telescopes and later for atmospheric distortions, the Hartmann sensor can be used as an X-ray wavefront metrology tool (Mayo & Sexton, 2004 ▸)^2^ by cutting a regular grid of small holes, spaced at known intervals (say *x*_*i*_ where *i* is the hole index), in a mask and then recording the shadow image on a detector, which is placed a small distance downstream of the mask.

Provided that each hole can be matched with each shadow image, the angle made between them, 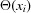 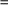 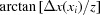 (in one dimension), is equal to the average direction of propagation of that part of the wavefront passing through each hole 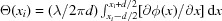, where Δ*x*(*x*_*i*_) is the observed displacement along *x* of the *i*th shadow, *z* is the distance between the mask and the detector, and *d* is the hole width.

With a suitable interpolation routine, Θ(*x*) can be estimated from the set of Θ(*x*_*i*_) and the phase profile can be obtained up to a constant with
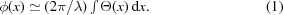
One limitation of this technique is that the resolution obtained is limited by the spacing between each hole in the mask. For example, Mercère *et al.* (2006 ▸) used a Hartmann sensor in an active optic system with a grid of 75 × 75 holes over a 10 × 10 mm area, whereas the CCD detector had a 1024 × 1024 grid of pixels over a 13 × 13 mm area. Thus the Hartman sensor had a resolution 10.5 times worse than the CCD detector.

**Figure 2:**
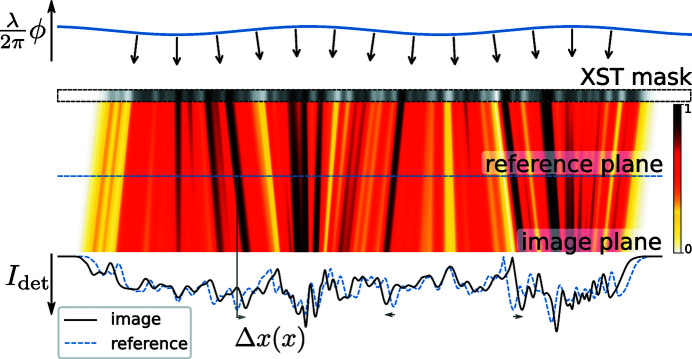
Illustration of the X-ray speckle tracking (XST) principle. Top: as in Fig. 1 ▸. Middle: as in Fig. 1 ▸, with the binary mask replaced by a random phase/absorption mask (dashed outline). Bottom: sub-regions of the measured shadow image (solid black line) are compared with the reference shadow image (dashed blue line) to determine displacements (black arrows).

The maximum density of the holes in the grid is limited. This is because the task of uniquely matching each shadow image with each hole becomes more difficult as the hole density is increased – a problem that is easier to appreciate in two dimensions. In 2012, Bérujon and co-workers realized a simple yet elegant solution to this problem, one that allowed for an arbitrarily fine grid of ‘masks’ with a resolution and sensitivity limited only by the CCD pixel array and the signal-to-noise ratio (Bérujon, Ziegler *et al.*, 2012 ▸). Their solution, XST, is to replace the binary mask of identical holes with a thin random phase object, such as a diffuser, as shown in Fig. 2 ▸. Because the diffuser is random (in the sense that the modulation of the beam by the diffuser is both detailed and non-repeating over the relevant spatial frequencies of the image), the shadow from each sub-region of the diffuser is unique – encoded by the speckle pattern seen on the detector – so that one can therefore consider any point in the diffuser to be the centre of a virtual Hartmann hole. In this sense, the random object serves as a high-density fiducial marker for each of the light rays that pass from the reference or mask plane to the detector. Note that this approach requires a greater degree of beam coherence than the Hartman sensor, to the extent necessary to provide sufficient visibility of the speckles, so that each speckle pattern can be distinguished from its neighbour.

In the Hartmann sensor, it is assumed that the mask is well characterized, so that the shadow positions can be compared with their ideal positions, which are known *a priori*. However, since the mask is no longer a simple geometric object (in the sense that it is difficult to know *a priori* the modulation function of the mask with a sufficient degree of precision), it is now necessary to record a reference image of the mask with which to compare the distorted image.

In addition to measurements of a wavefront’s phase, the XST principle can be extended to incorporate phase imaging of samples. This can be achieved by recording an image of the wavefront with the diffuser (acting as a mask) in the beam path – this image is called the ‘reference’ image. Then, another image is recorded with an additional sample (the one to be imaged) placed in the beam path, in addition to the diffuser – this is referred to simply as the ‘image’. Here the relative displacements between the ‘reference’ and the ‘image’ are due not to the phase profile of the wavefront, which affects both images equally, but to the phase profile of the sample transmission function.

The following two XST imaging configurations were suggested by Bérujon *et al.*, one for imaging samples and the other for wavefront metrology:

(i) in the differential configuration a speckle image is recorded with and without the addition of a sample, and

(ii) in the absolute configuration a speckle image is recorded at two detector distances with respect to the mask.

In (i), the relative motion of speckles reveals the local phase gradient of the sample in the beam, whereas in (ii), the total wavefront phase is recovered and this is therefore useful for characterizing X-ray beamline optics (this is the configuration shown in Fig. 2 ▸). Of course, it is still possible to characterize beamline optics in (i) (just not *in situ*) by placing the optical element in the sample position. This approach has been useful, for example, in measuring the phase profile of compound refractive lens systems (Bérujon, Wang & Sawhney, 2012 ▸) but is impractical for larger systems such as Kirkpatrick–Baez mirrors.

Since the proposal by Bérujon *et al.* there have been a number of substantial improvements: see for example the extensive review by Zdora (2018 ▸). For example, Zanette *et al.* (2014 ▸) developed a method where a diffuser is scanned so as to obtain a number of reference/image pairs at different diffuser positions. This step can add a great deal of redundancy, which improves the angular sensitivity of the method and even allows for multi-modal imaging of the sample when employed in the differential configuration. In subsequent publications, this approach has been termed the unified modulated pattern analysis (UMPA) method (Zdora *et al.*, 2017 ▸, 2018 ▸).

In the absolute configuration, where the reference and image have been recorded at two detector distances, the smallest resolvable angular displacement (the angular sensitivity) is given by the ratio of the effective pixel size, which is the smallest resolvable displacement of a speckle (including effects such as fringe visibility, finite pixel size, beam coherence and noise), to the distance between the reference and image planes: Δθ = *d*/Δ*z*. Therefore, the best accuracy is obtained by maximizing the distance between the reference and image planes. However, for highly divergent wavefields, as would be produced (for example) by a high-numerical-aperture lens system, there arises an unavoidable trade-off between the wavefront sampling frequency and the angular sensitivity. In this situation the ideal location of the image plane is as far downstream of the lens focus as is required to fill the detector array with the beam, as this maximizes the wavefront sampling frequency. In order to minimize Δθ (maximize Δ*z*) one should then place the reference plane as close as possible to the beam focus. But in this plane, the footprint of the beam on the detector may be much smaller than that in the image plane because of the beam divergence. This leads to a poorly sampled reference, as only a few pixels will span the wavefront’s footprint. Therefore, the smallest resolvable speckle shift will be larger than that obtainable by plane-wave illumination, by a factor proportional to the beam divergence.

Realizing this, Bérujon *et al.* (2014 ▸) devised an XST technique, X-ray speckle scanning (XSS), that relies on small displacements of the XST mask between acquired images. No reference is required and the diffraction data are recorded in a single plane. This enables the sampling frequency to be maximized by placing the detector such that the divergent beam fills the pixel array. Without a reference, however, the speckle locations in one image are instead compared with the locations observed in neighbouring images. As the speckle displacements in each image are proportional to the phase gradient, the differentials of the speckle locations between images are proportional to the second derivative of the phase; thus this approach can be viewed as a wavefront curvature measurement. The achievable angular sensitivity is now proportional to the step size of the mask, which can be substantially smaller than the effective pixel size. Interestingly, this approach is similar in principle to the Wigner-distribution deconvolution approach described by Chapman (1996 ▸).

In the following section, we describe an approach that is similar in principle to the one described above:

(iii) ptychographic XST: shadow images are recorded as the mask/object is translated across the wavefront.

**Figure 3:**
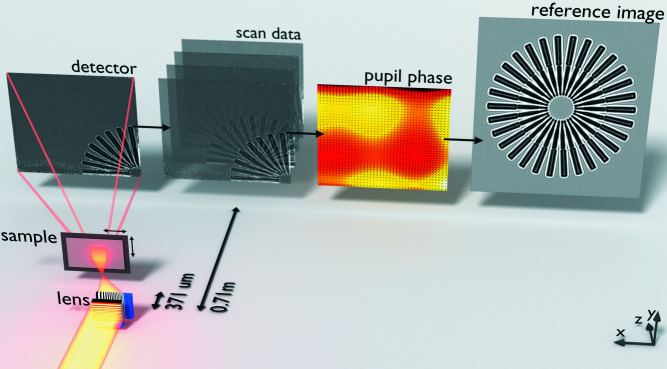
Illustration of the ptychographic XST method. The beamline illumination was focused (off-axis) in two dimensions by two linear focusing lenses, with numerical apertures of 0.015 (horizontal) and 0.014 (vertical). The Siemens star sample was placed 371 µm downstream of the focal plane. Images were recorded on a CCD pixel array detector 0.71 m downstream of the focus. The scan data consist of 49 shadow images, recorded as the sample was translated across the beam profile. The wavefront phase and reference maps were refined iteratively.

In this method (see Fig. 3 ▸) the unknown object acts as both the imaging target and the speckle mask simultaneously. There is no special reference image; rather each image serves as a reference for all other images. Both the wavefront phase (without the influence of the object) and the object image (without the influence of wavefront distortions) are determined in an iterative update procedure. At each iteration, speckles^3^ in the recorded images are compared with the current estimate of the reference (in contrast to the XSS method). Images are recorded at a fixed detector distance and there is no trade-off between phase sensitivity and the wavefront sampling frequency, making this method suitable for highly divergent beams. Because the speckle displacements are compared between the image and the estimated reference, large angular distortions can be accommodated. This is advantageous because it allows for the sample to be placed very near the beam focus, where the phase gradients across the sample surface are largest and where the magnification factor allows for high imaging resolution and angular sensitivity.

## 3. The speckle tracking approximation   

In this section we describe the governing equation that relates the measured intensities in each image and the reference in terms of the wavefront phase. For monochromatic light, in the Fresnel diffraction regime the image formed on a detector placed a distance *z* downstream of an object is given by
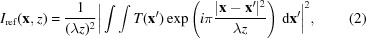
where *T*(**x**) represents the exit-surface wave of the light in the plane *z* = 0. For plane-wave illumination, under the projection approximation [see equation 2.39 of Paganin (2006 ▸)], *T*(**x**) also represents the transmission function of the object.

Now let us suppose that, rather than plane-wave illumination, the object is illuminated by a wavefront with an arbitrary phase (ϕ) and amplitude (*w* ^1/2^) profile given by *p*(**x**, 0) = *w* ^1/2^(**x**)exp[*i*ϕ(**x**)]. The observed intensity is now given by
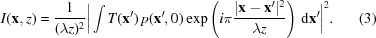



For XST-based techniques, the challenge is to relate the image (*I*) to the reference (*I*_ref_) via a geometric transformation. Here, the reference as defined in equation (3) represents an image of the sample, a distance *z* downstream of the sample plane, that is neither distorted by wavefront aberrations nor magnified by beam divergence. For the purposes of this section, *I*_ref_ could be a recorded image, but in subsequent sections we will see that this image can be estimated from a set of distorted images.

Note that at this point the mathematical description is rather general. For example, in the differential configuration of XST, *T*(**x**) would represent the wavefront generated by the diffuser in the plane of the object and *p*(**x**, 0) would represent the transmission function of the object. In what follows, however, we will continue to describe *T*(**x**) as the object or mask transmission function and *p*(**x**, *z*) as the X-ray beam profile (unmodulated by the object).

A common approach to this problem is outlined by Zanette *et al.* (2014 ▸). There, ϕ is expanded to first order, and *w* ^1/2^ to zeroth order, in a Taylor series about the point **x**:
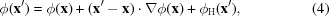


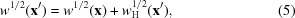
where ϕ_H_(**x**′) and 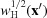 are the higher-order terms in the expansion. Now we have, for ϕ_H_ and 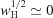,
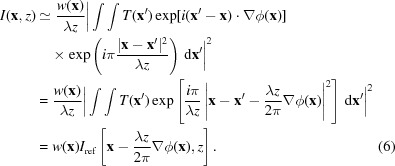
This confirms the intuitive assumption that the local gradient of ϕ at each position along the sample is converted into a lateral displacement of the speckles observed in the reference. Equation (6) serves well in the limit where ϕ_H_ and 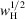 approach 0 and is employed in a number of XST-based techniques. For example, in the UMPA approach [see equation (9) of Zdora (2018 ▸)] the governing equation is given by 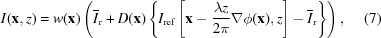
where 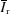 is the mean intensity of the reference pattern and *D*(**x**) is a term the authors refer to as the ‘dark-field signal’. This term is related to a reduction in fringe visibility due to fine features in *w*(**x**) and, in fact, serves as an alternative contrast mechanism when solved for in addition to the phase gradients. Putting this term aside by setting *D* = 1, one can see that equation (7) reduces to equation (6).

Given the restrictive nature of the approximations employed, however, it is not surprising that equation (6) quickly fails to serve as a valid approximation for larger phase gradients. To see this, let us consider a well known analytical solution to *I* in terms of *I*_ref_ called the ‘Fresnel scaling theorem’, which is described in, for example, Appendix *B* of Paganin (2006 ▸). Simply put, it states that

The projected image of a thin scattering object from a point source of monochromatic light is equivalent to a magnified defocused image of the object illuminated by a point source of light infinitely far away.

The derivation is rather simple and so we shall present it here using the current notation. Let us say that the image, *I*, is formed by the point source of illumination a distance *z*_1_ along the optical axis (the *z* axis) and that this distance is large enough that we can ignore intensity variations of the illumination across the sample surface, so that *w* ^1/2^(**x**) = 1. The probing illumination in the plane of the sample is then given by 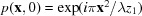. Substituting this into equation (3) and completing the square in the exponent, we have
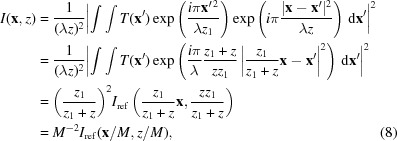
where the geometric magnification factor 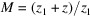 and *z*/*M* is the effective propagation distance (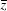). But according to equation (6) we would have 
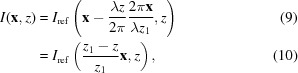
with a geometric magnification factor 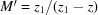, in contradiction to the result from the Fresnel scaling theorem. As expected, the results agree in the limit *z*_1_ → ∞, *i.e.* in the limit where the phase gradient approaches 0. Current formulations for XST based on equation (6) (in the absolute configuration) are expected to perform badly when the effective source distance, *z*_1_, approaches the propagation distance, *z*, or (in the differential configuration) when the sample transmission function departs significantly from the weak phase approximation.

In a notable departure from this approach, Paganin *et al.* (2018 ▸) have recently developed an alternative description of the speckle tracking approximation based on a ‘geometric flow’ equation:
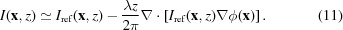
This approximation, which closely resembles the transport of intensity equation (Teague, 1983 ▸), has the remarkable property that ϕ may be determined analytically from a reference–image pair, thus permitting the rapid and simple processing of large tomographic data sets. This approach also assumes small and local distortions of the reference and is, therefore, ill suited as an approximation for larger phase gradients. For example, substituting the quadratic phase for a diverging wavefield, 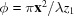, into equation (11) yields 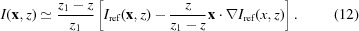
This corresponds to a geometric magnification factor of 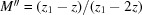, once again in contradiction to the analytical result 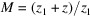.

To see this more clearly, let us examine the exact result of equation (8) in the limit where *M* → 1. First, we set 1/*M* = 1 + *m*, so that *m* → 0 as *M* → 1. Then we expand *I*_ref_(**x**/*M*, *z*/*M*) to first order in a Taylor series about **x**: 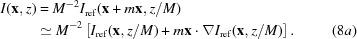
Comparing the above equation with equation (12), we have 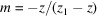. Solving for the geometric magnification factor yields 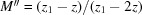 as above.

**Figure 4:**
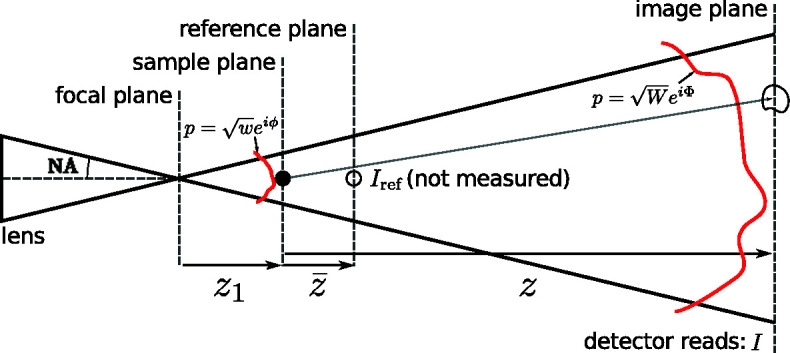
Schematic diagram for a hypothetical projection imaging experiment. The illuminating beam propagates from left to right and the solid black lines indicate the boundaries of the illumination wavefront. The sample is depicted as a small black filled circle in the sample plane and as a black circle in the reference and image planes. The red lines depict the illumination’s wavefront in the sample and image planes, which are not merely related by transverse magnification. The distorted shape of the circle in the image plane represents possible distortions of the speckle produced by the sample and the transverse phase gradients of the illumination.

Remarkably, with only a minor modification to the speckle tracking formula in equation (6), a second-order expansion of the phase term can be accommodated in the Fresnel integral, leading to the ‘speckle tracking approximation’: 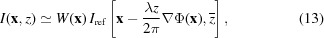


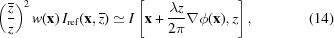
where ∇ϕ and ∇Φ are the transverse gradients of the illuminating wavefield phase in the sample and image planes, respectively (without the influence of the object), and *w* and *W* are the intensity profiles of the illuminating wavefield in the reference and image planes, respectively. In Fig. 4 ▸ we show a diagram for a hypothetical PXST imaging experiment. This diagram shows the lens, focal, sample, reference and image planes. The reference would have been measured by plane-wave illumination in the plane indicated. A point that is not illustrated in the diagram is that both the image and the reference exhibit propagation effects, such as Fresnel fringes. We note, once again, that the speckle tracking approximation above applies to more imaging geometries/modalities than that displayed in Fig. 4 ▸.

Equations (13) and (14) are reciprocal statements of the same approximation and choosing between them is a matter of convenience depending on the desired application. We note here that this approximation makes a distinction between the phase gradients in the sample and image planes, whereas it is common to assume that they are similar or related by a lateral scaling factor (magnification). This distinction is not important in cases where the separation between these two planes and the beam divergence is small, but becomes critical for highly magnified imaging geometries or long propagation distances. This approximation is not as strong as the ‘stationary phase approximation’ (Fedoryuk, 1971 ▸), which links coherent propagation theory with geometric optics, although the principles used to derive this result are similar. The derivation is straightforward and self-contained but lengthy, and may be found in Appendix *A* [App appa].

Equations (13) and (14) possess two beneficial properties for the current analysis: they relate the image and its reference via a geometric transformation and they are consistent with the Fresnel scaling theorem. In fact, the Fresnel scaling theorem is a special case of the above approximations when *w*(**x**) = 1 and 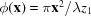. Evaluating equation (14) for these values of *w* and ϕ and using 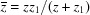 we have 
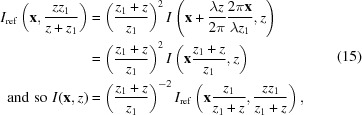
which yield the correct magnification and scaling factors, in agreement with equation (8). Similarly, we can evaluate equation (13) using 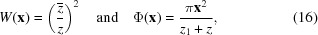
where these values for the illumination’s wavefront in the plane of the detector follow from the Fresnel approximation for a point source placed a distance *z* + *z*_1_ upstream and from flux conservation of the beam when *w*(**x**) = 1 in the sample plane.

Evaluating equation (13) yields 
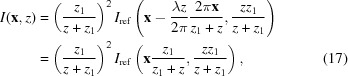
which is, once again, in agreement with equation (8).

In general, for arbitrary ϕ, the phase curvature of the illumination may vary in direction, as is the case (for example) in an astigmatic lens system, and also with position in the image. Thus, the magnification is also position dependent and directional: 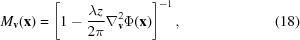
where ∇_**v**_Φ(**x**) is the directional derivative of Φ(**x**) along the unit normal vector **v**.

Given the extended validity of equation (13), we suggest that the following modification to the UMPA equation [equation (7)] will achieve better results: 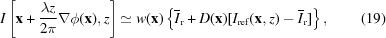
where 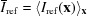, or, using the notation of Zdora (2018 ▸), 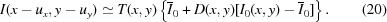
We also note that, although Paganin *et al.*’s geometric flow algorithm [equation (11)] is a poor approximation for larger distortion factors (large *M*), it may be a more general physical description in the limit *M* → 1. As the authors note, the term ∝ ∇*I*_ref_ · ∇ϕ in the expansion of equation (11) accounts for speckle translations that arise from strong intensity gradients of the reference, *i.e.* that are not generated from ∇ϕ alone.

## 4. Limits to the approximation   

The second-order speckle tracking approximation of equations (13) and (14) is subject to the following approximations:

(1) 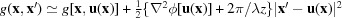,

(2) 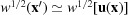,

(3) 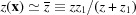,

where these are additional to the approximations necessary for the paraxial approximation to hold, 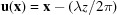
 ×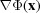 and 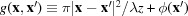. In general, these approximations hold best for smooth wavefront amplitudes *w* ^1/2^, predominantly quadratic phase ϕ and large spatial frequencies of the object.

Here, we examine the speckle tracking approximation, in one dimension, for the imaging geometry depicted in Fig. 4 ▸ and with parameters corresponding to a typical experiment utilizing X-ray multilayer Laue lenses. For this example we choose that the illumination is formed by a lens with a hard-edged aperture and with the sample placed at two possible distances from the focal plane, *z*_1_ = 500 and 10 µm. The lens has a numerical aperture of NA = 0.01 and the detector is placed in the far field of the probe and the sample, with *z*_1_ + *z* = 1 m. This imaging geometry leads to an effective propagation for plane-wave illumination that is nearly identical to the distance from the focus to the sample (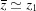). The wavelength is 10^−9^ m. The sample has a Gaussian profile so that 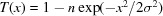, where *n* = 1 − *i* was chosen arbitrarily and would be proportional to the sample thickness and the deviation from unity of the refractive index and σ is the sample width, set to one of 0.15 or 0.01 µm below. The Fresnel number is thus 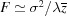.

The wavefronts in the sample and image planes were simulated using the discrete form of the Fresnel diffraction integral. The illumination’s wavefront in the image plane is given by *p*(*x*, *z*) = *c* *W* ^1/2^(*x*)exp[*i*Φ(*x*)], where *c* is a complex pre-factor that does not depend on *x*, *W* ^1/2^(*x*) was calculated numerically and Φ is almost quadratic, with 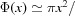 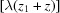. Note that ϕ(*x*), the phase profile of the illumination in the sample plane, is not given by 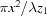 as would be the case for a point source of light (*i.e.* for NA → ∞). This is because the hard edges of the aperture produce Fresnel fringes that progress from the edge of the wavefront to the focal point at *x* = 0 as one moves from the image to the focal plane.

To test the validity of the speckle tracking approximation, we compare these simulated Fresnel images with those formed by evaluating equation (3). In this case equation (13) can be evaluated analytically with
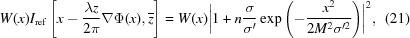
where
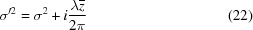
and 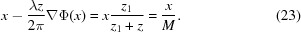
In order to arrive at the above result, we have assumed that Φ is purely quadratic across the wavefront, but this approximation has not been used when simulating the image according to Fresnel diffraction theory.

In Appendix *B* [App appb], we suggest a suitable criterion for the speckle tracking approximation to hold for this imaging geometry based on the second criterion above,
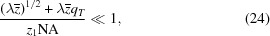
where *q*_*T*_ = 1/*X* is the spatial frequency corresponding to full period features of size *X*. This criterion holds for features within the plateau of the illumination profile.

**Figure 5:**
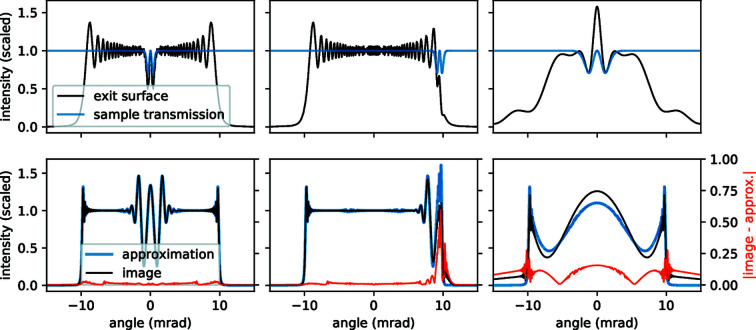
Comparison between the images formed according to Frensel diffraction theory and the speckle tracking approximation. In the left and middle columns, the sample has a σ width of 0.15 µm and is placed 500 µm from the focus. In the left column the sample is centred in the beam profile, whilst in the middle it has been shifted to the edge. In the right column the sample has a σ width of 0.01 µm and is placed 10 µm from the focus. First row: the exit-surface-wave intensities formed by illuminating a small Gaussian object with divergent illumination (black line). The intensities have been scaled by the factor 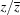. The sample transmission amplitudes are shown in blue. The angles along the *x* axis are given by 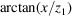 and match those of the second row. Second row: the intensity of the wavefront in the image plane (black line) and the images formed by the speckle tracking approximation (blue line). The fractional differences are shown in red. The angles are given by 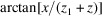.

In the first column of Fig. 5 ▸, we have placed the sample in the centre of the illumination profile. Here, the left-hand side of equation (24) evaluates to 0.8 and one can see that the fractional differences between the image and the approximation are small compared with that of the middle column. There, the sample has been shifted to the edge of the illumination profile, where the slope of the illumination amplitude is large. This leads to a breakdown of the second condition {*w* ^1/2^(*x*′) ≃ *w* ^1/2^[*u*(*x*)]}, and indeed the discrepancy between the approximation and the image is largest near the edge of the pupil region and slowly reduces for features closer towards the central region.

In the right column of Fig. 5 ▸, the sample is smaller, with a σ value of 0.01 µm, and has been moved closer to the focal point. The left-hand side of equation (24) now evaluates to 11.0. As expected, this increase from 0.8 in the first column to 11.0 in the right column corresponds to an increasing discrepancy between the speckle tracking approximation and the image. This image is in the transition region between the near-field and far-field diffraction regimes. Clearly, features in the diffraction outside of the holographic region, where *W* ^1/2^ ≃ 0, are not represented at all by the approximation.

In both the second and third examples shown here, the errors in the speckle tracking approximation are dominated by the error in the approximation *w* ^1/2^(*x*′) ≃ *w* ^1/2^[*u*(*x*)]. This is not surprising given that the zeroth-order expansion of *w* ^1/2^(*x*′) about *u*(*x*) is a much stronger approximation than the second-order expansion of ϕ(*x*′) about *u*(*x*) (both approximations are necessary to arrive at the speckle tracking formula).

The increased quality of projection images due to smoother illumination profiles was one of the principle motivations behind Salditt and collaborators’ efforts to develop an X-ray single-mode waveguide, in order to improve their tomo-holographic imaging methods [see for example Krenkel *et al.* (2017 ▸)].

## 5. Reconstruction algorithm   

**Figure 6:**
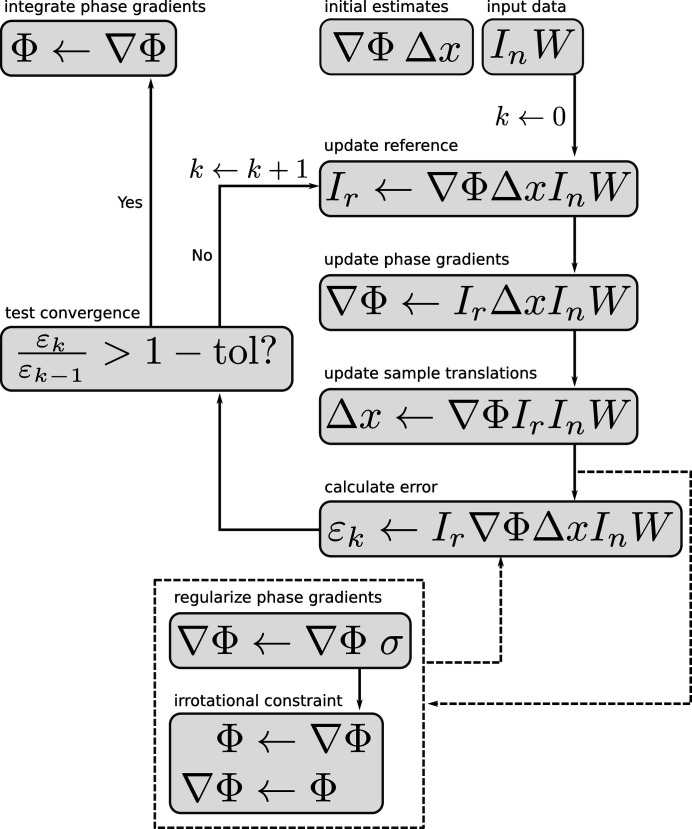
Flow diagram for the PXST iterative refinement cycle. A left arrow (←) represents an update of the item on the left given the items to the right of the arrow. The dashed line arrows represent optional paths in the algorithm. Each step in the diagram corresponds to an equation in the main text: ‘update reference’ to equation (27), ‘update phase gradients’ to equation (28), ‘update sample translation’ to equation (29), ‘calculate error’ to equation (26), ‘regularize phase gradients’ to equation (30), and finally the ‘irrotational constraint’ and ‘integrate phase gradients’ steps to equation (31).

In this section we describe the steps necessary to recover estimates for Φ(**x**) and *I*_ref_(**x**) from a series of *N* measurements of the kind depicted in Fig. 3 ▸, where each recorded image on the detector corresponds to a translation of the sample in the transverse plane by Δ**x**_*n*_ (here *n* is the image index). The refinement cycle is illustrated in Fig. 6 ▸. According to the speckle tracking approximation of equation (13), the geometric relationship between the recorded images *I*_*n*_(**x**) and the unrecorded reference *I*_ref_(**x**) is given by 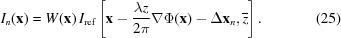
Translating the sample by Δ**x**_*n*_ along the *x* axis leads to a corresponding translation of the reference, because the convolution integral in equation (2) possesses translational equivariance.

To recover estimates for Φ(**x**) and *I*_ref_(**x**), we choose to minimize the target function 
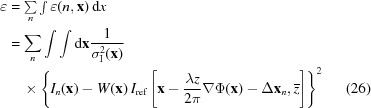
in an iterative update procedure with respect to ∇Φ(**x**) and (as needed) Δ**x**_*n*_, subject to
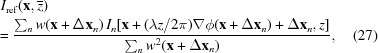
where 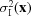 is the variance of the recorded intensities at each detector pixel, such that 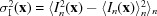. In fact equation (27) is the analytical solution for the minimum of 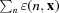 with respect to *I*_ref_(**x**) but for 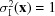. The reason we have set 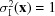 for the reference update is that, in this way, the reference is formed preferentially from parts of the image with larger intensities and thus will not be unduly affected by detector noise. This is also the update procedure that is often employed in single-mode ptychographic reconstructions [see for example equation (7) of Thibault *et al.* (2009 ▸)].

The update for ∇Φ(**x**) is given by 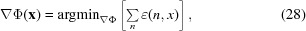
while holding *I*_ref_(**x**) and Δ**x**_*n*_ constant. Here argmin_∇Φ_ means ‘the argument of the minimum’ with respect to ∇Φ, which is to say, the ∇Φ that gives rise to the minimum of 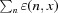. The minimization is performed by evaluating 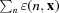 for possible value of ∇Φ(**x**) within a pre-defined search window.

The update for Δ**x**_*n*_ is given by
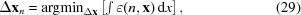
while holding *I*_ref_(**x**) and ∇Φ(**x**) constant. Once again, the minimization is performed by evaluating the possible value of Δ**x**_*n*_ within a pre-defined search window.

Additionally, it is often desirable to regularize ∇Φ(**x**) during the update procedure (especially for the first few iterations), according to
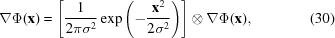
where ⊗ is the convolution operator and σ is a regularization parameter that can be reduced as the iterations proceed.

Once the iterative procedure has converged, the phase profile of the illumination [Φ(**x**)] can be recovered from the gradients [∇Φ(**x**)] by numerical integration. For this we follow the method outlined in the supplementary section of Zanette *et al.* (2014 ▸). Let us label the final value of the phase gradients by δ(**x**) ≡ ∇Φ(**x**). The procedure is then given by 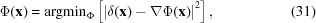
where ∇Φ(**x**) is evaluated numerically and the minimization is performed via the least-squares conjugate gradient method.

The fact that Φ is given by the numerical integration of ∇Φ suggests a further constraint that could be employed in the update procedure. As noted by Paganin *et al.* (2018 ▸), ∇Φ will be irrotational if Φ is continuous and single valued because ∇Φ is given by the gradient of a scalar field. This follows from the Helmholz theorem, which states that any field can be written as the sum of a gradient and a curl. Since we know that ∇Φ is, by definition, the gradient of Φ, then the curl must be zero: ∇ × ∇Φ = 0. In the work of Paganin *et al.*, this condition is automatically satisfied by the solution. Here, however, we must incorporate this as a separate constraint. An irrotational field **f** is one that satisfies
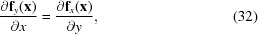
where **f**_*x*_(**x**) and **f**_*y*_(**x**) are the *x* and *y* components of the vector field, respectively. To ensure that ∇Φ is irrotational, one need only apply the numerical integration in equation (32) followed by numerical differentiation as needed during the update procedure. If this condition is not enforced, then the degree to which the recovered ∇Φ is irrotational can be used as a measure of the fidelity of the result.

Numerical considerations for the implementation of this iterative update procedure, in addition to the source code developed to implement the PXST algorithm, have been published online (see https://github.com/andyofmelbourne/speckle-tracking).

The algorithm presented here is by no means the only approach to solve for the phase gradients and the reference. Indeed, similar problems emerge in many areas of imaging such as computer vision (Demirci *et al.*, 2006 ▸), medical imaging (Thirion, 1998 ▸) and military targeting applications (Kechagias-Stamatis *et al.*, 2018 ▸). In magnetic resonance imaging, the process of identifying the distortions that relate an image to its reference is often termed the ‘image registration’ problem and generating the reference from a set of distorted views is termed ‘atlas construction’. ‘Diffeomorphic image registration’ algorithms are popular in that field, many of which are based on Thirion’s demons and log-demons algorithm (Thirion, 1998 ▸; Lombaert *et al.*, 2014 ▸). This approach has been employed in the context of XST by Berto *et al.* (2017 ▸) to recover the phase gradients from an image/reference pair. Others in the XST field use correlation-based approaches, where the geometric mapping between a small region of the distorted image and the reference is determined by the point which provides the greatest correlation coefficient (Zdora, 2018 ▸). The approach outlined in this work was employed because of its simplicity and ease of implementation. However, it seems likely (in the authors’ view) that one or more of the other approaches mentioned above could be adapted to the current problem in order to produce superior results.

### 5.1. Example reconstruction   

Here we provide a brief example of a PXST reconstruction from a simulated 1D data set. This example is not intended as a realistic simulation of an actual experiment: see Morgan *et al.* (2020 ▸) for experimental results in two dimensions. Rather, it serves as a simple illustrative check on the basic principles of PXST.

**Figure 7:**
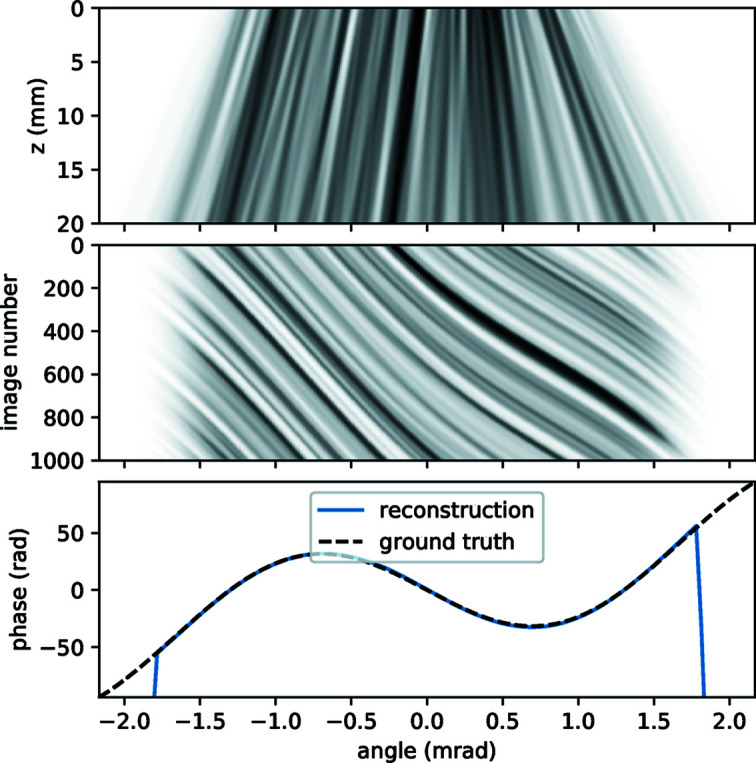
Top: intensity of the wavefront propagating from the sample (*z* = 0) to the detector plane (*z* = 20 mm). The linear colour scale ranges from 0 (white) to the maximum value (black). Middle: stack of the 1D images recorded as the sample is scanned across the wavefield (to the right). The colour scale is the same as in the top panel. Bottom: reconstructed and input phase aberrations in the detector plane. See text for further details.

The simulated sample is similar to that shown in Fig. 2 ▸. It was constructed in Fourier space with a Gaussian intensity profile and random phases at each pixel. The real-space object is thus complex valued, so that rays passing through the sample will be both absorbed and deflected in angle. The intensity of the illumination profile, in the plane of the detector, was formed by setting *W* equal to a top hat function filtered with a Gaussian kernel. This filter produces a smooth tapered fall-off in the intensity near the edges of the beam that helps to avoid aliasing artefacts during numerical propagation of the wavefront. The phase profile, Φ, was constructed with the quadratic function π*x* ^2^/[λ(*z*_1_ + *z*)], where λ = 1.2 nm (1 keV), *z* = 20 mm and *z*_1_ = 40 mm, so that the focal plane of the illumination is upstream of the sample in the top panel of Fig. 7 ▸ by a distance that is twice the sample-to-detector distance. This leads to an average magnification factor of 1.5. In addition to this, a sinusoidal phase profile was added to the phase in order to simulate the result of aberrations in the lens system; this can be seen as the dashed black line in the bottom panel of Fig. 7 ▸.

The intensity of the wavefront, *I*(*x*, *z*), propagating from the exit surface of the sample to the detector plane is shown in the top panel. Upon close inspection, one can see that the intensities in the plane of the detector are non-trivially related to those in the exit surface of the sample. As such *I*(*x*, 0) cannot be constructed from *I*(*x*, *z*) by a scaling in *x* (magnification) or indeed by any geometric mapping. We make the point again that in PXST the ‘reference’ is not the sample transmission profile; rather, it is the intensity profile one would have observed on a detector placed a distance 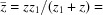 13.3 mm downstream of the sample illuminated by a plane wave. It is the geometric mapping between the reference (not the sample transmission) and the recorded images that is used to reconstruct the phase profile of the illumination.

The advantage of this 1D example is that one can visualize the entire data set in a single 2D image. In the middle panel of Fig. 7 ▸ the 1D images formed on the detector, as the sample is scanned across the wavefield, are displayed as an image stack. Along the vertical axis is the image number and the horizontal axis is in angle units, which are the angles made from the point source to each pixel in the image. It is seen that this image stack consists of a series of lines that appear to flow towards positive angles as the image number increases. These are the features in the image that can be obviously tracked through the stack. In this representation, the gradients of the lines at each diffraction angle are proportional the local wavefront curvature. For example, at a diffraction angle of ∼−0.75 mrad, the wavefront aberrations have a negative curvature and so features at this point in the wavefield are demagnified with respect to the mean. At a diffraction angle of ∼0.75 mrad, the opposite is true (with a greater magnification) and the line gradients are shallow with respect to those at ∼−0.75 mrad. In addition to variations in the geometric magnification, the wavefront aberrations also locally adjust the effective propagation distance of the speckles. This is a non-geometric effect and (unlike the local variations in the magnification) is not accounted for by the PXST reconstruction algorithm. For the current example, we have deliberately set the aberrations such that the local magnification and effective propagation distance vary by a significant fraction across the wavefield. This allows for their effect to be clearly observed in the simulated data, but also leads to some errors in the phases.

The reconstructed phase profile, after 30 iterations of the PXST algorithm, is shown as the blue line in the bottom panel of Fig. 7 ▸. The constant, linear and quadratic components of the phase (or pedestal, tilt and defocus terms, respectively) have been removed prior to display, to allow the sinusoidal aberration profile to be clearly visualized. Near the edges of the illumination, *W* ≃ 0 and the phases could not be determined (as expected). Apart from this, the differences between the ground truth and reconstructed phase profile (0.1 rad r.m.s. error) are too small to see in this plot but are still much greater than the theoretical lower limit of ∼0.0001 (this limit is defined in Appendix *D* [App appd]) – owing to the strength of the aberrations (as described in the previous paragraph).

## 6. Discussion and conclusion   

We have presented a modified form of the speckle tracking approximation, valid to second order in a local expansion of the phase term in the Fresnel integral. This result extends the validity of the speckle tracking approximation, thus allowing for greater variation of the unknown phase profile and for greater magnification factors when the wavefield has a high degree of divergence (such as that produced by a high-numerical-aperture lens system) or, when imaging a sample in the differential configuration of XST, allowing for greater phase variation across the transmission function of the sample (such as that produced by a thick specimen). We suggest that this approximation can be used, with little modification, in many of the existing XST applications and suggest such a modification for the UMPA approach.

We have also presented the PXST method, a wavefront metrology tool capable of dealing with highly divergent wavefields (like XSS), but unlike XSS, the resolution does not depend on the step size of the sample translations transverse to the beam. Coupled with a high-numerical-aperture lens, PXST provides access to nanoradian angular sensitivities as well as highly magnified views of the sample projection image. With a suitable scattering object, which in this case is the sample itself, a minimum of two images are required, although more images will improve robustness and resolution.

We must emphasize that it is only the projection image of the sample that is recovered. The phase and transmission profile of the sample must be inferred from the projection image via standard techniques (Wilkins *et al.*, 2014 ▸). This is in contrast to other methods that provide multiple modes of imaging of the sample, such as the transmission, phase and ‘dark-field’ profiles. What distinguishes PXST from these methods is that the sample image is obtained in addition to the wavefield phase in the absolute configuration of XST: that is, both can be obtained from a single scan series of the sample.

A further application of this method is to use it as an efficient prior step to Fourier ptychography, by recording images out of focus. The recovered illumination and sample profiles can be used as initial estimates for a Fourier ptychographic reconstruction. Experimentally, this additional step can be achieved simply by moving the sample towards the focal plane of the lens. In some cases, this additional step would not even be required, so that speckle tracking followed by ptychography could be performed on the same data set.

For experimental results utilizing the PXST method, see Morgan *et al.* (2020 ▸). These results are based on a campaign of measurements for the development of a high-numerical-aperture wedged multi-layer Laue lens systems.

## Figures and Tables

**Figure 8:**
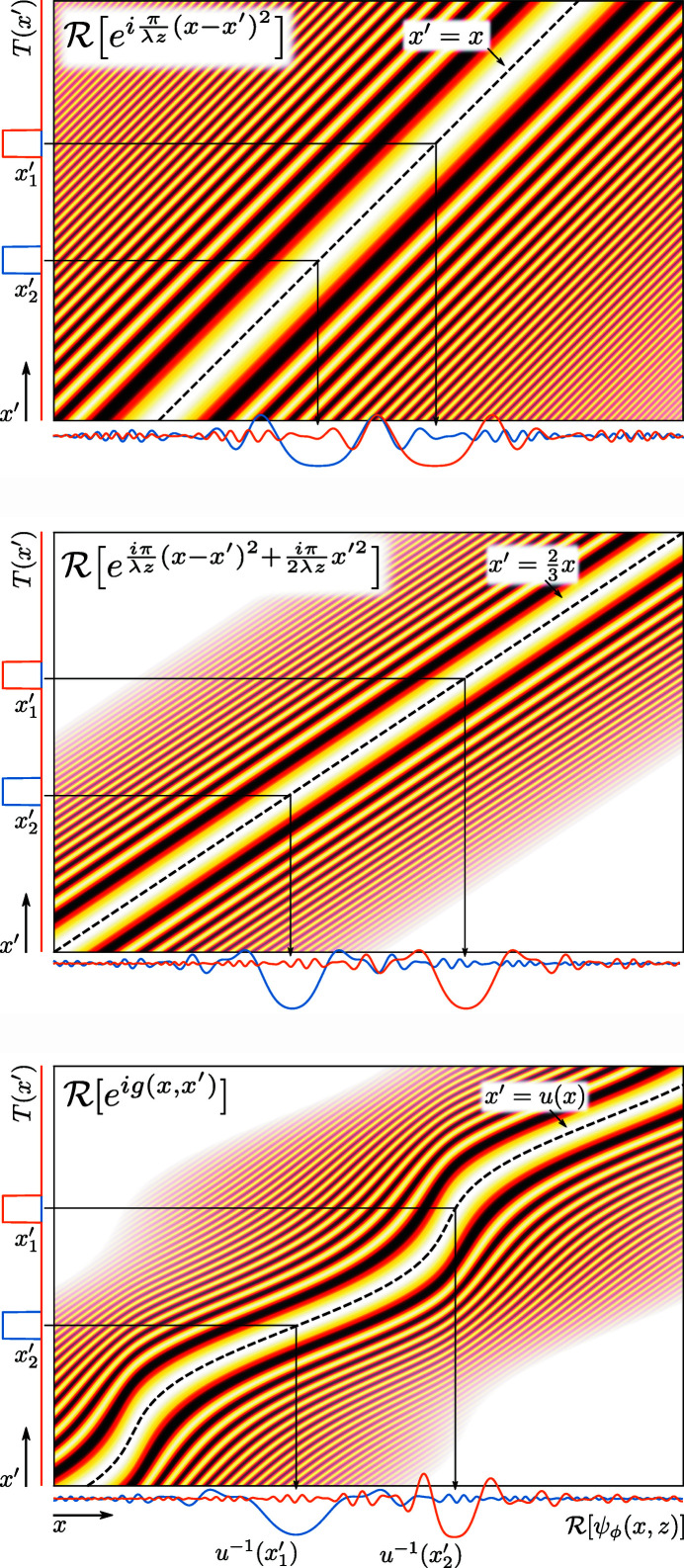
Illustration of the Fresnel integral, with a modulating phase term, for two top-hat functions. The colour maps display the real part of the exponential term, 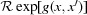, with the same colour scale as in Fig. 1 ▸. The alpha channel of the colour scale has been increased in regions where the spatial frequency of the exponent approaches the pixel size, so as to make transparent pixels that would otherwise be aliased. Phase terms that are constant with respect to *x* have been subtracted before display. These terms would not affect the final intensity of the image and removing them more clearly shows the line *x*′ = *u*(*x*) where 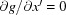. See text for further details.

**Figure 9:**
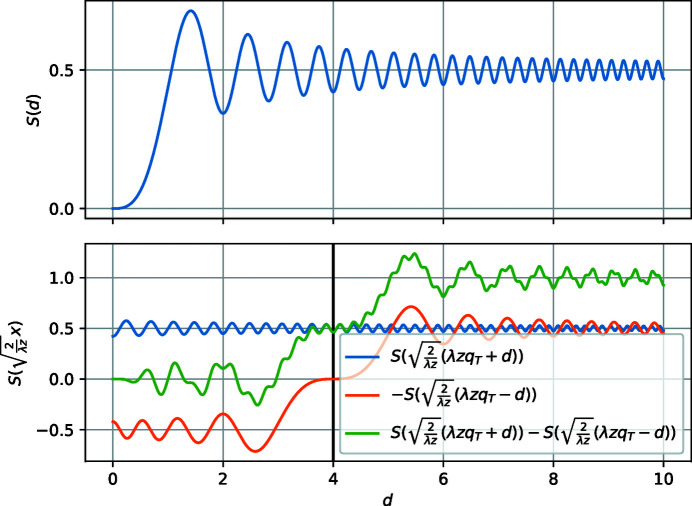
First row: the Fresnel integral *S*. Second row: the terms ∝ ψ(*x*, *z*; *q*_T_) in equation (78). For these plots, 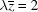 and the vertical black line is at 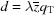.

**Table 1:** Symbols

*I*_*n*_(**x**, *z*)	*n* ^th^ recorded image
*I*_ref_(**x**, *z*)	Reference projection image of the sample
Δ**x**_*n*_	Displacement of sample in transverse plane
*T*(**x**)	Transmission function of the quasi-2D sample
*z*_1_	Source-to-sample distance
*z*	Sample-to-detector distance
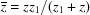	Effective propagation distance
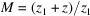	Geometric magnification factor
λ	Wavelength of radiation
σ_eff_	Smallest resolvable speckle displacement in the plane of the detector
*i*	(−1)^1/2^
**a** · **b**	Dot product between vectors **a** and **b**
*p*(**x**, 0) = *w* ^1/2^(**x**)exp[*i*ϕ(**x**)]	Illumination wavefront in the sample plane; *w* and ϕ are the intensity and phase, respectively
*p*(**x**, *z*) = *W* ^1/2^(**x**)exp[*i*Φ(**x**)]	Illumination wavefront in the detector plane; *W* and Φ are the intensity and phase, respectively
**x** ≡ (*x*, *y*)	Transverse coordinate
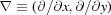	Transverse gradient operator

**Table 2:** The PXST method

Governing equation	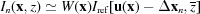	See Section 3 and Appendix *A* [App appa]
	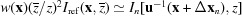	Reciprocal form for the above equation; **u** ^−1^ is the inverse of **u**
Target function	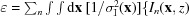 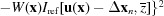	Equation (26) in Section 5; to be minimized with respect to *I*_ref_, ∇Φ and Δ**x** *_n_*
Geometric mapping	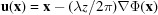	See equation (54) in Appendix *A* [App appa]
	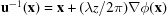	Reciprocal form for the above equation; see equation (37) in Appendix *A* [App appa]
Imaging geometry	See Fig. 3	Described in Section 2
Iterative update algorithm	See Fig. 6	Described in Section 3
Angular sensitivity	ΔΘ_ϕ_ = σ_eff_/*z*	In the plane of the sample; see equation (123)
	ΔΘ_Φ_ = σ_eff_/*zM*	In the plane of the detector; see equation (126)
Phase sensitivity	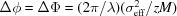	Sample/detector plane; see equation (130)

## Footnotes

2 In this work, an array of refractive elements was used rather than a grid of mask holes.

3 In this article we use the word ‘speckle’ loosely, to mean any localized diffraction feature recorded by the detector. Indeed, our method could just as well have been referred to as ‘ptychographic X-ray *feature* tracking’.

## Appendix A Derivation of the speckle tracking approximation

The Fresnel integral of equation (2) is often referred to as a point projection mapping. This is because, when the Fresnel number is ≫1, the dominant contributions to the integral typically arise from values of the sample transmission, *T*(*x*′), around the point *x* (*i.e.* for *x*′ ≃ *x*). At this point, the phase term π(*x* − *x*′)^2^/λ*z* has a spatial frequency *q*_F_ = (*x* − *x*′)/λ*z* ≃ 0. For *x*′ far from *x*, the phase term causes the integrand to oscillate rapidly between ±*T*(*x*′). If *T*(*x*′) is bandwidth limited, with a maximum spatial frequency of (say) , then, for a sufficiently large |*x* − *x*′|,  and successive oscillations of the integrand, caused by the phase term, will occur at roughly the same values of *T*(*x*′) and will thus cancel each other in the integration.

However, in the Fresnel integral of equation (3), the modulation of *T*(*x*′) by  has generated an additional phase term, and we would now expect the dominant contribution to *I*(*x*, *z*) from *T*(*x*′) to arise at values of *x*′ for which the integrand is smooth. To simplify the analysis, let us gather the phase terms of the Fresnel exponent and the incident illumination into a global phase factor

so that the complex amplitude of the Fresnel integral becomes^4^

Note that the global phase term *g*(*x*, *x*′), does not contain any contribution from the phase of the transmission function *T*(*x*′). Without any prior knowledge of this phase term, our smoothness condition becomes

For now, we will define *u* ^−1^ as the solution to this equation for *x* given *x*′, so that

*u* ^−1^ is the functional inverse of *u*, which is yet to be defined. So, equation (3) now represents the point projection mapping *x*′ → *u* ^−1^(*x*′), rather than *x*′ → *x*.

This point is illustrated in Fig. 8 ▸ with *w* ^1/2^(*x*′) = 1 for three different values of ϕ(*x*′). On the vertical axis of each panel, we plot two real top-hat functions representing possible values for *T*(*x*′). In the two-dimensional domain of the integrand, *T*(*x*′) is constant along the *x* axis. The Fresnel integral is performed by extruding *T*(*x*′) along the horizontal axis, multiplying by  and then integrating along the vertical axis. The real part of this integral is illustrated along the horizontal axis of each panel. We can see that the centroids of the features, before and after the Fresnel integral, follow the point projection mapping delineated by the dashed line *x*′ = *u*(*x*), which is defined by the condition . In the top panel ϕ(*x*′) = 0, leading to *x*′ = *u*(*x*) = *x*. This corresponds to Fresnel propagation with plane-wave illumination. Therefore, the separation between the top-hat functions in the sample plane is equal to the separation between the ‘speckles’ produced by each top-hat function. In the second panel ϕ(*x*′) = π*x*′^2^/(2λ*z*), corresponding to divergent illumination that would arise from a point source of illumination, or an ideal lens system with an infinite numerical aperture. Here *x*′ = *u*(*x*) = 2*x*/3 and, consistent with the Fresnel scaling theorem, this leads to both a geometric magnification (the speckles are separated by a greater distance than the top hats) and a change in the effective propagation distance (which can be observed in the more rapid oscillation of the exponential term). In the final panel, a sinusoidal phase term has been added to the phase from the middle panel. Here *u*(*x*) does not have a simple form and both the effective propagation distance and the magnification vary with position along the *x* axis.

This suggests the following modification to the approach outlined by Zanette and co-workers: instead of expanding ϕ(*x*′) and *w* ^1/2^(*x*′) about *x* in equation (3), we should shift this expansion about the point *x*′ = *u*(*x*). In this way, the Taylor series expansion will be most accurate over the domain of the integrand that contributes most to the integral. The *N*th-order Taylor series expansions of *g*(*x*, *x*′) and *w* ^1/2^(*x*′) about *x*′ = *u*(*x*) are given by

Evaluating equation (38) for *N* = 1 and equation (39) for *N* = 0, we have

where the *n* = 1 term in the expansion of *g* is zero by construction. With *g* ≃ *g*_1_ and , equation (34) becomes

Unfortunately, the above expression completely fails to capture the physics upon which XST methods are based, *i.e.* the geometric mapping between *I* and *I*_ref_ defined by ϕ. In our attempt to improve the accuracy of the speckle tracking approximation, the first-order expansion of *g* about *x*′ = *u*(*x*) no longer depends on *x*′. Thus the integral over *x*′ in equation (34) has reduced to the term .

With the above result in mind, let us try the following approach:

Instead of expanding ϕ(*x*′) to first order and *w* ^1/2^(*x*′) to zeroth order about the point *x*′ = *x*, expand *g*(*x*, *x*′) to second order and *w* ^1/2^(*x*′) to zeroth order about the point *x*′ = *u*(*x*).

This approach leads to

where, once again, the *n* = 1 term for *g*(*x*, *x*′) is zero by construction in equation (37). Substituting equations (40) and *w* ^1/2^(*x*′) ≃ *w* ^1/2^[*u*(*x*)] into (35) then completing the square in the exponent we can recast the Fresnel integral in the following form:

where we have defined *z*(*x*) as

One can interpret *z*(*x*) as the propagation distance required to locally reproduce the diffraction features in ψ(*x*, *z*) had the illumination been plane wave [*i.e.* ϕ(*x*′) = 1].

We remind the reader that the ‘ref’ subscript refers to the wavefront that would have been formed with plane-wave illumination, with *p*(*x*, 0) = 1. Here we define ψ_ref_ as the complex amplitudes corresponding to the Fresnel integral in equation (2):

Now ψ(*x*, *z*) can be related to ψ_ref_(*x*, *z*) (where *I*_ref_ = |ψ_ref_|^2^) by the substitutions *x* → *u*(*x*) and *z* → *z*[*u*(*x*)], yielding

So far we have avoided a more explicit definition of the geometric mapping factor *u*(*x*); it is currently defined by its inverse in equation (37). A more meaningful definition can be obtained by the following consideration. Setting *T*(*x*′) = 1, equation (41) represents the propagation of the incident beam through free space in the absence of the sample, so that

Here we have defined

and *W*(*x*) and Φ(*x*) are, respectively, the intensity and phase profiles of the undisturbed beam in the plane of the detector.

The benefit of this calculation is that it provides an interpretation of the mapping function *u*(*x*) in terms of the phase gradient of the illumination in the *z* plane. To see this, we first explicitly evaluate Φ(*x*) in terms of the incident phase profile ϕ(*x*). Substituting *x*′ = *u*(*x*) into equation (33), we have

Using the definition for *u* ^−1^(*x*) in equation (37), we can then evaluate

Taking the derivative of both sides of equation (49) with respect to *x* yields

With this equality and the definition for *u* ^−1^(*x*) in equation (37), one can now verify that the following equality holds:

But since *u*[*u* ^−1^(*x*)] = *x* we can identify *u*(*x*) with

Furthermore, we can evaluate *z*[*u*(*x*)] in terms of Φ(*x*) by making use of the following relation:

where we have used the expression for *z*(*x*) in equation (42). Then, taking the derivative with respect to *x* of both sides of equation (53),

and rearranging, we obtain

Inserting equations (47), (54) and (57) into equation (44) we can then write

or, the inverse relationship,

This formulation of the projected image separates the effects of the geometric and propagation-based distortions induced in the detected image by phase variations in the incident illumination. The geometric distortions are captured by the term (λ*z*/2π)∇Φ(*x*) and the change in the fringe structure of a feature by the term *z*_Φ_(*x*).

For the purposes of the current work, we will ignore variations in the fringing terms *z*(*x*) and *z*_Φ_(*x*) across the wavefield and use instead the constants

〈∇^2^ϕ(*x*)〉_*x*_ and 〈∇^2^Φ(*x*)〉_*x*_ are the mean phase curvatures of the illumination in the plane of the sample and the detector, respectively. Under the Fresnel approximation, they can be defined in terms of the effective source distance from the sample or detector planes. If *z*_1_ is the distance between the entrance surface of the sample and the effective source point, then

Using the above expressions, one can now verify that

With the above approximations for *z*(*x*) and *z*_Φ_(*x*) and taking the mod square of equations (58) and (59), we arrive at the 1D speckle tracking approximation:

In two dimensions, the constant prefactor to the Fresnel integral in equation (34) is 1/(−*i*λ*z*) [rather than 1/(−*i*λ*z*)^1/2^ in one dimension]. This leads to an altered expression for *W*:

Additionally,  is now defined by the average wavefront curvature over the 2D transverse plane:

where the Laplacian operator is now also over the 2D plane, . If one accepts the approximation  from the beginning, then the analysis presented here in one dimension can be repeated in two by following the above steps, first along the *x* axis and then along the *y* axis. The result of this procedure is

## Appendix B Derivation of the region of validity of the speckle tracking approximation

In Appendix *A* [App appa], it was shown that the Fresnel integral of *T*(*x*) modulated by *p*(*x*, 0),^5^

can be approximated by

subject to the approximations

(1) ,

(2) ,

(3) ,

where these are additional to the approximations necessary for the paraxial approximation to hold, *p*(*x*, 0) = *w* ^1/2^(*x*) × exp[*i*ϕ(*x*)], *p*(*x*, *z*) = exp(2π*iz*/λ)*W* ^1/2^(*x*)exp[*i*Φ(*x*)], *u*(*x*) = *x* − , *z*_1_ is the effective distance between the light source and the sample plane, and   .

### b1. Requirements

In this section we shall determine the requirements for each of these three approximations to hold.

#### b1.1. The first approximation

Let us assume for the moment that approximations 2 and 3 are valid. In this case, it is sufficient to require that the Taylor series expansion of *g*(*x*, *x*′) is valid within the interval of convergence of the Fresnel integral in equation (71).

In Appendix *A* [App appa], we have intuited that the expansion of *g*(*x*, *x*′) need only be valid for values of *x*′ satisfying *q*_*g*_(*x*, *x*′) < *q*_*T*_, where *q*_*g*_(*x*, *x*′) are the spatial frequencies of *g* and *q*_*T*_ is the maximum spatial frequency of *T*. For values of *x*′ outside of this region, successive oscillations of the integrand will occur at roughly the same magnitude [±*T*(*x*′)] with a net zero contribution to the integral.

So, let us first examine the domain of *x*′ over which the expansion is valid. The Lagrange error bound for the Taylor series expansion of *g*(*x*, *x*′), about *x*′ = *u*(*x*), sets a limit on the magnitude of the residual:

where

With the above expression, we can identify the interval 2*d*_ϕ_ of *x*′ about *u*(*x*), such that *x*′ : *u*(*x*) − *d*_ϕ_ → *u*(*x*) + *d*_ϕ_, for which the magnitude of the residual |*R*_*N*_| is below some threshold level of tolerance. So, for *N* = 2,

and

we have

where *E*_2_ is the Lagrange error bound for the second-order expansion of *g* and *E*_tol_ is the maximum tolerable error (which has been implicitly defined so as to absorb the factor of 6).

Now we determine the minimum interval  required for the Fresnel integral in equation (71) to converge near to its true value.

Let us expand *T*(*x*′) as a Fourier series, such that , where  is the complex amplitude of the *q*th full-period spatial frequency of *T*(*x*′) corresponding to features of extent *X* = 1/*q*.

By the superposition principle, we can select the highest spatial frequency of *T* such that  for |*q*| > *q*_*T*_ and examine the radius of convergence of the integral in equation (71) for :

where  is given by the value of *d* for which the above integral has converged within an acceptable error margin. This integral can reduced to the following form:

where the function *E* is known as the Euler or Cornu spiral and the ‘⋯’ represent terms that are constant with respect to the integration variable *v*. The Euler spiral can be constructed in the complex plane in terms of the Fresnel integrals *C* and *S*:

As both *C* and *S* approach their limit of  as *x* → ∞ with equal rapidity, we shall examine only the imaginary part of *E* for convenience.

In Fig. 9 ▸ we plot the imaginary part of the indefinite integral of equation (78) (shown in green in the second row) as a function of *d*. We can see here that for *d*   the integral oscillates about 1 and that the amplitude of these oscillations is dominated by the  term (shown in orange).

The extrema of *S*(*x*) are given by

and therefore

The extrema of  are therefore located at

So, the residual for the integral in equation (78) is proportional to 1/2 − *S*[(2*m*)^1/2^]. Therefore, the integer *m* will serve as a measure of convergence for the integral and we can require that . Finally, combining equations (76) and (83) we have, for ,

The above inequality can be seen as a limit for the phase variation  or, for a given , as a limit on the smallest features that will be resolved (*q*_*T*_ = 1/*X*) according to the approximation in equation (71). Setting, *m* ^1/2^ = 1, multiplying both sides of equation (84) by  and raising both sides by the third power, we have the condition

#### b1.2. Approximation 2

Assuming for now that approximations 1 and 3 are valid, we require that *w* ^1/2^(*x*′) ≃ *w* ^1/2^[*u*(*x*)] is valid within the interval |*x*′ − *u*(*x*)| < *d*_*w*_, and that this interval is larger that , which is the interval about *x*′ = *u*(*x*) over which the integral in equation (71) will converge. Making use, once again, of the Lagrange error bound we have

The requirement  leads to

#### b1.3. Approximation 3

Assuming that approximations 1 and 2 are valid, the governing equation becomes

where we have used  and restored *z*_Φ_(*x*) in place of  in equation (71). This approximation, that , is thus valid in the limit where the residual term

approaches zero. The conditions under which ψ(*x*, *z*_1_) ≃ ψ(*x*, *z*_2_), in the Fresnel diffraction regime, are well known: the requirement is that the Fresnel number *F* = *X* ^2^/λ|*z*_2_ − *z*_1_| ≫ 1. In this case, , which depends on *x*. To generalize this requirement across the entire wavefront, we thus require that

or

where σ(*z*) is the standard deviation of the effective propagation distance given by  and  is the Fresnel number for features of size *X* propagating a distance . Under this condition, features of size *X* (after correcting for the geometric distortions) will produce the same image on the detector regardless of their transverse position along the wavefront.

This condition can be expressed as a constraint on the phase profile of the beam. Using the definitions for *z*_Φ_(*x*) [equation (57)] and  [equation (61)] we have

Using the above equation we have that approximation 3 is valid in the limit

### b2. Limit on the defocus for an ideal lens

The illumination formed by an ideal lens, with a hard-edged aperture, has a distinct form (see for example Fig. 4 ▸). Within the plateau of the wavefront the intensity oscillates about a mean value, while the phase profile is approximately quadratic: ϕ ≃ π*x* ^2^/λ*z*_1_. In this case, approximation 2, that *w* ^1/2^(*x*′) ≃ *w* ^1/2^[*u*(*x*)], is the most onerous of the three. In the above analysis, we had used the Lagrange error bound to estimate the maximum distance along the wavefront (|*x*′ − *x*|) for which this approximation will hold. But for the present case, this estimate is over-bounded given its general nature. Instead, we propose that an acceptable condition for this approximation is that *w* ^1/2^(*x*′) ≃ *w* ^1/2^[*u*(*x*)] will remain valid for |*x*′ − *x*| < *z*_1_NA, where NA is the numerical aperture of the lens and *z*_1_NA is approximately equal to the half-width of the plateau. Thus we replace  with *d*_*w*_ = *z*_1_NA and equation (87) becomes

where this condition applies for features within the plateau of the illumination.

## Appendix C Uniqueness

### c1. Pedestal and tilt terms are unconstrained in the illumination phase profile

In general, the solution to the target function in equation (26) does not constrain terms proportional to 1, *x* and *y* in the recovered phase profile. These terms are sometimes referred to as the ‘pedestal’ and ‘tilt’ components of the pupil function. To see this, consider a phase profile Φ′ = *c* + **d** · **x** + Φ, where Φ corresponds to the true phase profile in the plane of the detector and where *c* and **d** are constants. This leads to the phase gradients ∇Φ′ = **d** + ∇Φ. Substitution into equation (25) yields

independent of the pedestal term. Also, the tilt in the phase profile has produced a shift in the reference, which is generally not detectable unless the position of a feature in the object is known *a priori* with respect to the detector.

Alternatively, we could have absorbed the term  as a constant offset in the sample translation vectors . We note that the tilt terms are typically unconstrained in speckle tracking techniques, since it is common to allow for an overall offset in the sample or detector positions.

### c2. Speckle patterns of sufficient density are necessary, but not sufficient, for a unique solution to exist

Clearly, in the extreme case where no speckles are recorded the phase is completely unconstrained, so that

and for any Φ′. This condition could also be reached in the limit where the fringe visibility of the speckle pattern approaches zero. The requirement for adequate fringe visibility is a point which is emphasized by Zanette *et al.* (2014 ▸) as well as many others in the field (Zdora, 2018 ▸). Of course, the above condition could also be reached for any sub-domain of **x**. If, for example, *I*_*n*_(**x**′) = *W*(**x**′) for all *n*, then the phase terms are unconstrained at the points **x**′. This suggests a more general (necessary) condition for a unique solution to exist: that a speckle of sufficient contrast must be observed at least once at each position in the image. As an example, the Hartmann sensor discussed in Section 2 does not satisfy this condition and, as such, it is necessary to interpolate values of Φ between the mask holes, rendering the method insensitive to high-order aberrations that lead to rapid variations in ΔΦ. Note that this is not always an issue, especially in cases where the low-order aberrations of the wavefront are of primary concern, such as when we wish to correct them by some means, or when the low-order aberrations are dominant and dominate the imaging performance of the optic.

In another extreme, the phase is also unconstrained for *N* = 1, when only a single image has been recorded. This is because the unknown *I*_ref_ can be adjusted to accommodate any Φ′:

and for any Φ′. This situation has arisen because the observed speckles are modelled as a function of ∇Φ and *I*_ref_, both of which are refined in the PXST method. So if a given speckle is observed only once, at a location **x**′, and the phase gradient at **x**′ is not otherwise constrained, then multiple solutions for ∇Φ and *I*_ref_ exist. The above two considerations suggest the more general (necessary) conditions for a unique solution to exist:

A speckle of sufficient contrast must be observed at least once at each position in the image, and this speckle must be observed at least twice and at different positions in the image.

Note that the above constraint does not require that every speckle must be observed more than once.

This is the ‘speckle density condition’ alluded to in the title of this section. The easiest way to satisfy this condition is to use a sample that produces a dense high-contrast array of speckles on the detector, such as a diffuser. With such a sample, the above constraint may be satisfied with just two images (*i.e.* for *N* ≥ 2), provided that the sample step size is not greater than half the illuminated region of the sample along the direction of the step.

However, it is possible for a unique solution to exist even when the sample produces only a single observable speckle in each image. In this case, the above condition can be satisfied by scanning the sample such that this speckle is observed at each point in the image. This is a far less efficient means for wavefront sensing than using a diffuser. But this generality allows for nearly any object, such as the Siemens star in Fig. 3 ▸ or a Hartmann mask, to be used as a wavefront sensing device.

### c3. Ambiguities can arise from unknown sample positions

If the sample translation vectors (Δ**x**_*n*_) are unknown, then there exists a family of solutions to equation (25), with each solution corresponding to a set of translation vectors related by an affine transformation. Consider a set of translation vectors Δ = **x**_0_ + **A** · Δ**x**_*n*_, where **x**_0_ is an overall offset and the dot product is between the 2 × 2 linear transformation matrix **A** and the true sample translation vectors. As described previously, any overall offset in the translation vectors generates a corresponding offset in the reference and a tilt term in the recovered phases. So, neglecting the offset term, we generate this family of solutions by the substitution Δ**x**_*n*_ = **A** ^−1^ · Δ into equation (25):

where

and

If, on the other hand, the true sample translation vectors are given by the input values of Δ**x**_*n*_, but with small random offsets of mean 0, then the true solution for the phase and reference can be recovered from the retrieved values by removing the effect of any affine transformation that may have arisen during the reconstruction. This can be accomplished by minimizing  with respect to **A**, where  and  are the input and output values of Δ**x**_*n*_, respectively, then generating the corresponding solutions for ∇Φ′ and  from the above equations. This situation can arise, for example, from small relative errors in the translation of a stepper motor or from the pointing jitter of an X-ray free-electron laser pulse.

### c4. An unknown rotation of the sample stage axes with respect to the detector axes can be corrected

A common systematic error for the input sample positions is an overall rotation of the axes of the sample translation stages with respect to the pixel axes of the detector. In this case the linear transformation matrix reduces to the rotation matrix:

Here, we can make use of the fact that in general ∇Φ′ of equation (110) is not irrotational for θ ≠ 0 and **A** = **R**(θ). If (*u*, *v*) ≡ ∇Φ^out^ then we can require that the vector field **R** ^−1^ · (*u*, *v*) be irrotational. With **R** ^−1^ = **R**(−θ) and equation (32), we have

where the derivatives with respect to *x* and *y* are evaluated numerically. However, if the irrotational constraint was enforced during the reconstruction, then the recovered  is free of the erroneous rotation and no further analysis is required.

### c5. The raster grid pathology produces artefacts for lattice-like sample translations

A common problem encountered in ptychography is the ‘raster grid pathology’ (Thibault *et al.*, 2009 ▸). The raster grid pathology arises when reconstructing both the illumination and sample profiles from diffraction data acquired while the sample is scanned along a regular grid. In that case, the recovered illumination and sample transmission functions may be modulated by any function, so long as it is periodic on a lattice of points upon which all of the sample positions lie.

In many cases, the governing equation for a ptychographic reconstruction is given by

where  is the Fourier transformation operator over the transverse plane and represents the propagation of the exit-surface wavefront ψ_*n*_(**x**) ≡ *T*(**x** − Δ**x**_*n*_)*p*(**x**, 0) to the detector (in the far field of the sample). If the sample is translated along a regular grid, for example, with step size **d**, then Δ**x**_*n*_ = **n** · **d**, where the vector **n** = (*i*_*n*_, *j*_*n*_) is the 2D lattice index corresponding to the *n*th image (for integer *i*_*n*_ and *j*_*n*_). If we make the substitution *p*′(**x**, 0) = *f*(**x**)*p*(**x**, 0) into the above equation, then we have

and hence

if  for all **n**. The raster grid pathology can be avoided by ensuring that the sample scan positions lack any translational symmetry, *i.e*. by scanning the sample in non-regular patterns, for example in a spiral grid, or by adding a random offset to every grid position (Fannjiang, 2019 ▸). Given that equation (113) is just a special case of the Fresnel integral in equation (2) (from which the speckle tracking approximation is derived) it is natural to consider whether or not the same pathology applies here.

One can show that a similar pathology does indeed arise in the present case. Here, the illumination’s intensity is constrained during the reconstruction, so instead we make the substitution *p*′(**x**, *z*) = *p*(**x**, *z*)exp[*ig*(**x**)], which is equivalent to Φ′(**x**) = Φ(**x**) + *g*(**x**), into equation (25):

and hence

if  for all **n**. So, rather than modulating the reference with a periodic function, the pathology here creates a periodic geometric distortion of the reference.

## Appendix D Angular sensitivity and imaging resolution

### d1. The resolution of the reference is given by the demagnified effective pixel size

As discussed in Section 3, the Fresnel scaling theorem states that the projection image of a thin sample formed by a point-like source of coherent light produces a magnified and defocused image of the sample. Similarly, in PXST, the ‘reference’ is an idealized image that would have been formed if the illumination were plane wave (*i.e.* with a flat phase profile), the detector were placed a distance  from the plane of the sample, the detector extended over the entire illuminated region of the sample and the physical pixel size (σ_det_) were reduced by the magnification factor *M*. Assuming that the speckle tracking approximation holds, and that the aggregate signal-to-noise level is high, then the resolution of such an image is given by the demagnified effective pixel size of the detector (σ_ref_).

The effective pixel size (σ_eff_) can be much smaller than σ_det_ owing to sub-pixel interpolation, which is employed when registering a speckle across many images. We have found, as others have noted (Bérujon, Ziegler *et al.*, 2012 ▸), that sub-pixel interpolation can lead to a reduction in the effective pixel size by a factor of 10 or more depending on the point-spread function of the detector, the contrast of the speckles, the signal-to-noise ratio per image and the total number of images. On the other hand, effects such as the finite source size of the X-rays will tend to blur-out speckles and increase the effective pixel size.

Consider the imaging geometry depicted in Fig. 4 ▸, for an incoherent source of X-rays with a Gaussian angular distribution given by , where θ is the angle made by a ray pointing from the incoherent source point to the lens aperture. Then the image recorded in the detector plane will be given by

where ⊗ is the convolution operator, *f* is the focal length of the lens and *I*(**x**, *z*) is the intensity of the wavefront in the plane of the detector for an on-axis point source of light. Therefore, the observed speckles will be broadened by a factor σ_s_ *zf*/*z*_1_. In this regime, the effective pixel size is given by

where we have used the symbol δ_pix_ to represent the fractional reduction in the effective pixel size due to numerical interpolation. For example, with a physical pixel size of σ_eff_ = 50 µm, a fully coherent wavefield and δ_pix_ = 1, the demagnified pixel size for the example shown in Fig. 5 ▸ (left and middle columns) would be σ_ref_ = 25 nm and for the right column (with the sample 10 µm from the focus) σ_ref_ = 5 Å.

### d2. The sample position that maximizes the angular sensitivity of the wavefront, in the plane of the sample, depends on the source coherence width

The smallest resolvable angular deviation of the wavefront, in the plane of the sample, is given by the arctangent of the smallest resolvable displacement of a speckle over the distance between the sample and the detector pixel array:

where we have employed the small-angle approximation in the last equality. The smallest resolvable increment in the phase gradient is thus . We note that ΔΘ_ϕ_ is a lower bound on the angular sensitivity, since the achieved value will generally be larger than σ_eff_/*z* because of detector noise and/or photon counting statistics.

For the imaging geometry depicted in Fig. 4 ▸, the optimal position for the detector will be given by the furthest distance from the focus such that the footprint of the illumination is contained within the pixel array, as this maximizes the sampling frequency of the wavefield. This raises the question, how far should one place the sample from the focus in order to maximize the angular resolution (minimize ΔΘ)? To answer this, let us keep the focus-to-detector distance (*z*_t_) fixed, so that *z*_t_ = *z*_1_ + *z*, and minimize ΔΘ_ϕ_ with respect to *z*_1_. Inserting *M* = *z*_t_/*z*_1_ and equation (122) into equation (123) and minimizing yields

for

As the focus-to-sample distance is reduced, the magnification factor increases (improving the angular sensitivity), while at the same time the deleterious effects of the finite source size increase (deteriorating the angular sensitivity). The above value for *z*_1_ represents the optimal compromise between these two effects.

### d3. The sample position that maximizes the angular sensitivity of the wavefront, in the plane of the detector, is in the focal plane where the magnification is greatest

The angular resolution for the wavefield in the plane of the detector (ΔΘ_Φ_) is not, in general, the same as the angular resolution in the plane of the sample. This is because of the difference in extent between the effective pixel size and the demagnified effective pixel size due to divergent illuminating wavefields. ΔΘ_Φ_ is given by

For a fixed focus-to-detector distance, once again *z* = *z*_t_ − *z*_1_, and we have

An interesting feature of the above equation is that the effect of the source incoherence δ_pix_ *f*σ_s_/*z*_t_ does not vary with *z*_1_; as *z*_1_ is decreased the increase in the magnification factor leads to a corresponding decrease in the effective pixel size due to the finite source size, but this is exactly balanced by the reduction in the angle subtended by the demagnified effective pixel size. Therefore, the optimal position for the sample, in order to minimize ΔΘ_Φ_, is as close to the focal plane as possible. However, this can be a dangerous limit to approach, as the speckle tracking approximation will begin to break down as a result of the rapidly varying illuminating wavefield – at this point, it would be necessary to employ a fully coherent model for the wavefront propagation, for example, by switching to far-field ptychography. Additionally, it can be beneficial to increase the focus-to-sample distance for practical reasons; for example, increasing *z*_1_ provides a larger field of view of the sample in each image which aids in positioning the region of interest of the sample with respect to the illuminating beam and, typically, increases the speckle visibility. For these reasons, it can be beneficial to approach the limit where

Here *z*_1_ has been increased until the demagnified pixel size is approximately equal to the demagnified feature size one would observe from a point-like object owing to incoherence alone. This represents the transition between modes where ΔΘ_Φ_ is dominated by the detector pixel size (larger *z*_1_) and by the finite source size (smaller *z*_1_).

### d4. Although the angular sensitivities of the wavefields in the sample and detector planes differ, the phase sensitivities are equal and are both minimized by maximizing the magnification

The difference between ΔΘ_ϕ_ and ΔΘ_Φ_ may at first appear to be a curious asymmetry. However, the phase sensitivities in the planes of the sample and the detector are in fact equal for a given sample position. For divergent illumination, the angular distribution of the wavefield in the sample plane is larger than that of the wavefield in the detector plane by a factor of *M*. On the other hand, the sampling frequency of the wavefield in the sample plane is also larger by the factor *M*. So, when propagating uncertainties in the angular distributions to the integrated phase profiles, these two effects cancel.

Recall the relationship between the integrated phase profile and the angular distribution of the wavefield in equation (1). Uncertainties in the angular distribution are thus scaled by the step size in *x* after integration, so that

Therefore, with σ_ref_ = σ_eff_/*M*, we have that   and both are minimized by placing the sample as close as possible to the focal plane, as described in the previous subsection.
